# Application of the PM6 semi-empirical method to modeling proteins enhances docking accuracy of AutoDock

**DOI:** 10.1186/1758-2946-1-15

**Published:** 2009-09-11

**Authors:** Zsolt Bikadi, Eszter Hazai

**Affiliations:** 1 Virtua Drug Ltd, Csalogany Street 4C Budapest, Hungary

## Abstract

**Background:**

Molecular docking methods are commonly used for predicting binding modes and energies of ligands to proteins. For accurate complex geometry and binding energy estimation, an appropriate method for calculating partial charges is essential. AutoDockTools software, the interface for preparing input files for one of the most widely used docking programs AutoDock 4, utilizes the Gasteiger partial charge calculation method for both protein and ligand charge calculation. However, it has already been shown that more accurate partial charge calculation - and as a consequence, more accurate docking- can be achieved by using quantum chemical methods. For docking calculations quantum chemical partial charge calculation as a routine was only used for ligands so far. The newly developed Mozyme function of MOPAC2009 allows fast partial charge calculation of proteins by quantum mechanical semi-empirical methods. Thus, in the current study, the effect of semi-empirical quantum-mechanical partial charge calculation on docking accuracy could be investigated.

**Results:**

The docking accuracy of AutoDock 4 using the original AutoDock scoring function was investigated on a set of 53 protein ligand complexes using Gasteiger and PM6 partial charge calculation methods. This has enabled us to compare the effect of the partial charge calculation method on docking accuracy utilizing AutoDock 4 software. Our results showed that the docking accuracy in regard to complex geometry (docking result defined as accurate when the RMSD of the first rank docking result complex is within 2 Å of the experimentally determined X-ray structure) significantly increased when partial charges of the ligands and proteins were calculated with the semi-empirical PM6 method.

Out of the 53 complexes analyzed in the course of our study, the geometry of 42 complexes were accurately calculated using PM6 partial charges, while the use of Gasteiger charges resulted in only 28 accurate geometries. The binding affinity estimation was not influenced by the partial charge calculation method - for more accurate binding affinity prediction development of a new scoring function for AutoDock is needed.

**Conclusion:**

Our results demonstrate that the accuracy of determination of complex geometry using AutoDock 4 for docking calculation greatly increases with the use of quantum chemical partial charge calculation on both the ligands and proteins.

## Background

The role of in silico chemistry is emerging in drug design and discovery. In an effort to find lead compounds at lower cost and greater speed, computational chemistry methods have focused on developing fast and highly efficient molecular docking methods for virtual screening [1,2]. In recent years, progress has been made in developing docking algorithms that predict ligand binding to proteins and by now several docking programs are available such as AutoDock, [3,4], GOLD, [5,6], Glide, [7,8] and FlexX [9,10]. Among these, the AutoDock program was the most popular according to a recent study [11].

Molecular docking methods include the search in space for the energetically most favorable conformation of a protein-ligand complex and the scoring of the resulting geometries with respect to binding energy [1,12]. The production of the right docking pose and the scoring of the complex geometries are often treated as two separate problems. It should be noted however, that many docking programs use the scoring function in the process of finding the complex with lowest energy [13]; thus, scoring and geometry prediction should rather be treated as one problem and it can be assumed that minimizing the RMSD between predicted and experimentally determined complex geometries would lead to more accurate prediction of binding free energies at the same time.

In AutoDock 4 energy scoring function the calculation of pair-wise atomic terms includes evaluations for different secondary interactions, dispersion/repulsion, hydrogen bonding, electrostatics, and desolvation [14]. Thus, calculation of accurate partial charges on the ligand and the protein is expected to have a profound effect on both the docking conformation and on the energy score of the resulting complex, possibly leading to more accurate estimation of complex geometry and binding energy. There are several charge calculation methods which lead to significant differences in the partial charges assigned to the different atoms [15]. AutoDockTools program enables the user to use empirical charge calculations, Gasteiger or Kollman united charges. However, this charge calculation method has been shown to yield less accurate partial charges than semi-empirical methods [16]. Additionally, Gasteiger charge calculation [17] does not handle electrons, presenting a major flaw in the docking calculation of metalloproteins.

Moreover, in a recent study analyzing the effect of various charge models in docking results it was concluded that the quantum mechanical charge calculation method yielded significantly better docking results [15,18], both in terms of binding geometry and energy. It should be noted that in these studies only the ligand charges were calculated with the quantum mechanical method, while the protein charges were calculated with the Gasteiger-Hückel method. Still, semi-empirical charge calculation on the ligand was enough to yield more accurate docking results. Quantum mechanical polarization of the ligand also has been shown to greatly improve docking accuracy [19]. Illingworth and his colleagues [20] extended this method by calculating polarization not only on the ligands, but also on the target macromolecules using Amber charges [21]. However, those implementations involve the knowledge of the structure of the complex and iteration of quantum mechanical calculations and thus cannot be treated as a practical tool in docking [20]. Raha and Merz used semi-empirical QM based scoring function for predicting binding energy and binding mode of a diverse set of protein-ligand complexes [22]. The authors used a scoring function designed using semi-empirical QM Hamiltonians to discriminate between native and decoy poses generated from the program AutoDock 4. Recently, a newly developed semi-empirical PM6 method was introduced that corrects major errors in AM1 and PM3 calculations and is useful for semi-empirical charge calculations of small ligands as well as proteins [23]. Besides that, all main group elements and transition metals are parameterized in PM6 in MOPAC2009 software. Thus, using the PM6 method for assigning partial charges to both the ligand and the protein would have two main advantages i.e. docking of metalloproteins can accurately be handled and semi-empirical charge calculation is expected to yield more accurate docking results in general.

In the current study it was analyzed whether PM6 semi-empirical charge calculation on both the ligands and their host proteins increases docking accuracy in terms of complex geometry and binding energy using AutoDock 4 software. To the author's knowledge this is the first study where MOPAC2009 software is used for semi-empirical charge calculations on proteins systematically for preparing input files for docking calculations. 53 protein-ligand complexes were analyzed for which both crystallographic structure determination and binding data were available. The partial charges of the ligands and proteins were calculated using 1.) Gasteiger 2.) PM6 charge calculation methods and the ligands were docked using AutoDock 4 software back into their host proteins. The resulting complex geometries were analyzed for their RMSD as compared to the available X-ray structure and their binding energies as calculated by the AutoDock 4 scoring function (docking result defined as accurate when the RMSD of the first rank docking result complex is within 2 Å of the experimentally determined X-ray structure). Our results indicated that the use of the PM6 semi-empirical charge calculation method for assigning partial charges to both the protein and the ligand atoms greatly increases docking accuracy as compared to the Gasteiger charge calculation method (available in AutoDockTools) in terms of complex geometry.

## Results

In Table 1 structural and experimental data of the investigated protein-ligand complexes are summarized. The 53 complexes used in this study were all characterized by a resolution below 3.2 Å. The complexes were chosen partly from the AutoDock 3.0 calibration set [68], from a recently published paper examining different docking software [13] and from the core set of PDBbind Database [69]. The chosen structures possess structurally diverse ligands in complex with a heterogeneous collection of proteins (see Table 1). It should be noted that for some structures with lower resolution (although chosen from the AutoDock 3.0 calibration set), an RMSD-based comparison of docked versus experimental structure might not always lead to a meaningful result as partial occupancies might occur that are not reflected by a single ligand structure. Using this dataset, ligand and protein structures were setup using two different methods, (i), calculating Gasteiger charges on both the ligands and the proteins using AutoDockTools and (ii), calculating PM6 charges on both the ligands and the proteins using MOPAC2009 [70] on Docking Server (see Method section for details). Docking calculations were performed twice on the dataset (in case of both ligand and protein set up methods) and the results were then compared to the experimentally determined complex structures.

**Table 1:** Experimental data for the 54 protein-ligand complexes.

PDB code	Protein	Res (Å)	Ligand	Structure Ref.
1AI5	Penicillin acylase	2.4	m-nitrophenylacetic acid	[24]

1AJP	Penicillin acylase	2.3	2,5-dihydroxyphenylacetic acid	[24]

1AMW	Heat shock protein 90	1.9	ADP	[25]

1BGQ	Heat shock protein 90	2.5	radicicol	[26]

1CBR	Retionic acid binding protein	2.9	retinoic acid	[27]

1D3Q	Human thrombin	2.9	benzo [*b*]thiophene derivative	[28]

1D3T	Human thrombin	3.0	benzo [*b*]thiophene derivative	[28]

1DWB	a-Thrombin	3.2	benzamidine	[29]

1FLR	Immunoglobulin	1.9	fluorescein	[30]

1GNI	Human serum albumin	2.4	oleic acid	[31]

1HVJ	HIV-1 Protease	2.0	A78791	[32]

1HVR	HIV-1 Protease	1.8	XK263	[33]

1K4G	tRNA-guanine transglycosylase	1.7	quinazoline derivative	[34]

1KV1	p38 MAP kinase	2.5	pyrazol derivative	[35]

1LIF	Adipocyte lipid-binding protein	1.6	stearic acid	[36]

1M0N	Dialkylglycine decarboxylase	2.2	1-aminocyclo-pentanephosphonate	[37]

1M0Q	Dialkylglycine decarboxylase	2.0	S-1-amino-ethanephosphonate	[37]

1OLU	Branched-chain alpha-ketoacid dehydrogenase kinase	1.9	thiamin diphosphate	[38]

1Q8T	cAMP-dependent protein kinase	2.0	(R)-trans-4-(1-aminoethyl)-n-(4-pyridyl) cyclohexanecarboxamide	[39]

1RBP	Retinol-binding protein	2.0	retinol	[40]

1S39	tRNA-guanine transglycosylase	2.0	2-aminoquinazolin-4(3H)-one	[41]

1U33	Alpha-amylase	2.0	5-trihydroxy-6-hydroxymethyl-piperidin-2-one	[42]

1ULB	Purine nucleoside phosphorylase	2.8	guanine	[43]

1UWT	Beta-glycosidase	2.0	D-galactohydroximo-1,5-lactam	[44]

1X8R	3-Phosphoshikimate 1-carboxyvinyltransferase	1.5	phosphonate analogue	[45]

1XD1	Alpha-amylase	2.2	acarbose derived hexasaccharide	[46]

1YDT	cAMP-dependent protein kinase	2.3	n-[2-(4-bromocinnamylamino)ethyl]-5-isoquinoline	[47]

1ZC9	Dialkylglycine decarboxylase	2.0	pyridoxamine 5-phosphate	[48]

2ACK	Acetylcholinesterase	2.4	edrophonium ion	[49]

2BAJ	p38alpha Map kinase	2.3	pyrazol derivative	[50]

2BAK	p38alpha Map kinase	2.2	nicotinamid derivative	[50]

2CEQ	Beta-glycosidase	2.1	glucoimidazole	[51]

2CET	Beta-glycosidase	2.0	phenethyl-substituted glucoimidazole	[51]

2CGR	Immunoglobulin	2.2	N-trisubstituted guanidine	[52]

2CPP	Cytochrome P-450cam	1.6	camphor	[53]

2D3U	RNA-dependent RNA polymerase	2.0	non-nucleoside analogue inhibitor I	[54]

2D3Z	RNA-dependent RNA polymerase	1.8	non-nucleoside analogue inhibitor II	[54]

2FDP	Beta-secretase	2.5	amino-ethylene inhibitor	[55]

2G94	Beta-secretase	1.9	valinamide derivative	[56]

2GBP	D-galactose/D-glucose binding protein	1.9	glucose	[57]

2IFB	Fatty-acid-binding protein	2.0	palmitic acid	[58]

2IWX	Heat shock protein 82	1.5	synthetic macrolactone	[59]

2J77	Beta-glycosidase	2.1	deoxynojirimycin	[Gloster, to be published]

2J78	Beta-glycosidase	1.7	gluco-hydroximolactam	[Gloster, to be published]

2QFU	3-Phosphoshikimate 1-carboxyvinyltransferase	1.6	shikimate-3-phosphate	[60]

2QWB	Neuraminidase	2.0	sialic acid	[61]

2QWD	Neuraminidase	2.0	4-amino-Neu5Ac2en	[61]

2R04	Rhinovirus 14 coat protein	3.0	W71, antiviral agent	[62]

2XIS	Xylose isomerase	1.6	D-xylitol	[63]

2YPI	Triose phosphate isomerase	2.5	2-phosphoglycolic acid	[64]

3PTB	b-Trypsin	1.8	benzamidine	[65]

4HMG	Hemagglutinin	3.0	sialic acid	[66]

7ABP	Arabinose-binding protein	1.7	fucose	[67]

### Estimation of binding energies

**Figure 1:**
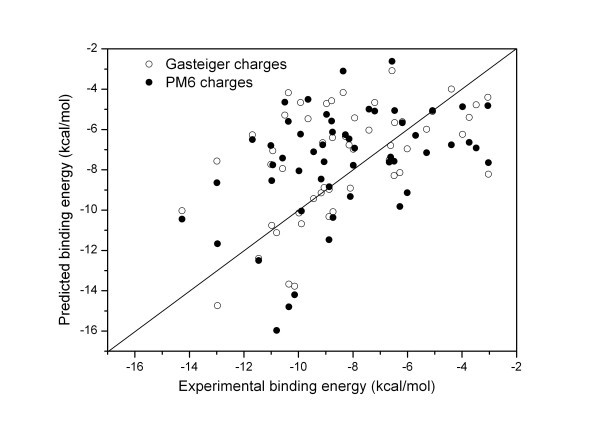
**Correlation between experimental and predicted binding energies using Gasteiger and PM6 charge calculations**.

Figure 1 shows the correlation between experimentally determined and predicted binding energies as calculated by AutoDock 4. The correlation between the predicted and observed binding energy is rather limited (correlation coefficient is about 0.51). In most cases the binding energy is underestimated by the prediction. It should be noted that the correlation coefficient increases by considering only the hits where the first rank result yielded an RMSD within 2 Å of the X-ray structure (correlation coefficient is about 0.60) in cases where Gasteiger partial charge calculation method is used. Thus, it can be concluded that good geometry prediction does contribute to accurate binding energy estimation. Compared to Gasteiger, PM6 had somewhat lower regression constant (R = 0.41 for all cases, R = 0.46 with an RMSD below 2 Å) in the docking studies (Figure 1). Thus, the change in the method of partial charge calculation even decreased the predicted total binding energy using the current AutoDock 4 scoring function. This result is not surprising considering the fact that AutoDock 4 scoring function was optimized using the Gasteiger charge calculation method.

### Geometry prediction in docking studies using Gasteiger charges on both ligand and protein atoms

The results of the docking calculations using Gasteiger charges on both the ligand and the protein are summarized in Table 2. Analyzing the first rank results, 28 of the 53 complexes resulted in an accurate docking result (lowest RMSD within 2 Å as compared to the X-ray structures). Considering the most populated cluster (and not the lowest energy) as a first rank result, 30 of the 53 dockings were able to successfully predict the experimentally observed binding mode. Among these successful predictions, 24 were found to be the lowest energy and most populated result at the same time. Docking calculations using Gasteiger charges resulted in 14 "dominant" (more than 60% of the dockings in the same cluster) first rank results in successful predictions. In 8 cases there were no accurate dockings (RMSD below 2.0 Å in any of the clusters) among the docking runs. The average RMSD of the first rank results was 2.34 Å (1.83 Å without outliers).

**Table 2:** Results of docking calculations using Gasteiger charges on both the ligand and the protein.

PDB code of the complex	RMSD of the first rank result as compared to the X-ray (Å)	Energy rank of the lowest RMSD hit	Frequency rank/frequency of the best geometry	Lowest RMSD (Å)	Estimated free energy of binding (kcal/mol)	Experimental binding energy (kcal/mol)
1AI5	1.21	**1**	**1/48**	0.65	-5.11	-5.08

1AJP	2.56	2	**1/47**	1.09	-4.40	-3.05

1AMW	1.68	**1**	**1/21**	1.59	-5.62	-6.19

1BGQ	1.03	**1**	**1/48**	0.71	-6.27	-11.69

1CBR	1.00	**1**	**1/51**	0.92	-7.94	-10.58

1D3Q	2.68	2	2/19	1.23	-10.33	-8.88

1D3T	2.29	2	2/20	1.08	-10.08	-8.73

1DWB	9.59	3	**1/51**	0.53	-6.24	-3.98

1FLR	0.60	**1**	**1/100**	0.53	-10.76	-10.98

1GNI	1.56	**1**	**1/49**	1.10	-7.73	-11.01

1HVJ	3.12	-			-10.03	-14.27

1HVR	0.65	**1**	**1/26**	0.54	-14.74	-12.97

1K4G	2.82	4	**1/30**	1.66	-6.98	-7.99

1KV1	0.64	**1**	**1/98**	0.56	-8.91	-8.10

1LIF	2.33	4	**1/21**	1.08	-5.47	-9.65

1M0N	3.91	**-**			-8.22	-3.03

1M0Q	2.24	5	33/1	1.46	-5.99	-5.30

1OLU	1.34	**1**	4/10	0.74	-6.96	-6.01

1Q8T	1.88	**1**	**1/77**	1.41	-8.29	-6.49

1RBP	0.84	**1**	**1/100**	0.56	-9.15	-9.17

1S39	4.29	2	2/11	0.32	-5.29	-10.50

1U33	2.49	**1**	2/14	1.73	-8.14	-6.28

1ULB	1.88	**1**	**1/100**	1.83	-4.66	-7.20

1UWT	2.36	**1**	**1/89**	0.87	-6.77	-8.14

1X8R	1.24	**1**	**1/80**	0.87	-4.17	-8.36

1XD1	2.92	**-**			-11.12	-10.80

1YDT	2.2	2	**1/30**	0.74	-10.14	-9.98

1ZC9	2.71	-			-4.00	-4.39

2ACK	3.96	8	8/1	1.07	-4.72	-8.97

2BAJ	0.68	**1**	**1/74**	0.42	-12.40	-11.46

2BAK	1.28	**1**	16/2	1.28	-13.78	-10.14

2CEQ	7.14	**-**			-4.66	-9.92

2CET	4.86	**-**			-7.06	-10.94

2CGR	0.87	**1**	**1/43**	0.50	-10.68	-9.89

2CPP	1.56	**1**	**1/100**	1.21	-6.35	-8.28

2D3U	0.87	**1**	**1/58**	0.83	-9.43	-9.44

2D3Z	4.68	3	2/20	0.69	-8.89	-9.06

2FDP	2.35	5	73/1	1.68	-13.67	-10.35

2G94	3.13	**-**			-7.57	-12.99

2GBP	0.99	**1**	**1/90**	0.92	-4.18	-10.37

2IFB	2.03	2	**1/45**	1.10	-6.03	-7.41

2IWX	0.88	**1**	**1/68**	0.55	-6.66	-9.11

2J77	1.9	**1**	**1/100**	1.85	-7.52	-6.67

2J78	0.83	**1**	2/11	0.83	-6.41	-8.75

2QFU	2.75	3	3/1	1.02	-6.29	-5.70

2QWB	4.38	13	6/5	1.17	-5.40	-3.74

2QWD	1.36	**1**	**1/54**	0.97	-6.80	-6.62

2R04	2.06	2	3/21	1.02	-8.97	-8.87

2XIS	1.20	**1**	**1/95**	1.15	-5.43	-7.94

2YPI	4.73	-			-3.09	-6.57

3PTB	1.35	**1**	**1/100**	0.51	-5.66	-6.47

4HMG	1.82	**1**	**1/22**	1.02	-4.78	-3.48

7ABP	2.15	2	2/13	1.64	-4.58	-8.78

	**Average: 2.34 without outliers (rmsd above 4 Å): 1.83**	**28 first hits**	**30 first hits**	**Average: 1.00**		

The average value of the RMSD between the lowest energy result and experimental structure and the total number of successful first rank predictions (based on lowest energy and highest cluster population, respectively) are indicated at the bottom of the table.

### Geometry prediction in docking studies using PM6 charges on both ligand and protein atoms

Docking calculations were performed for the same dataset, using the PM6 method for both protein and ligand setup [23]. Comparing partial charges on a selected atom, PM6 method gives higher absolute value for partial charges than the Gasteiger calculation. In the case of proteins, the absolute value of partial charges returned a value of about 1.6 times greater on average than in the case of Gasteiger charge calculation. i.e. the sum of the absolute value of partial charges was 628.8 in case of Gasteiger charge calculation, while it was 1014.2 using PM6 in case of the protein with PDB entry 4HMG; the sum of absolute values of partial charges was 354.1 with Gasteiger charge calculation and it increased to 570.8 with PM6 partial charge calculation in case of the protein with PDB code 1HVR. The ratio of absolute partial charge values using Gasteiger and PM6 calculation methods was found to be constant among the investigated proteins; Gasteiger/PM6 0.619 ± 0.020 (data not shown). In the AutoDock 4 scoring function, the sum of absolute partial charges effects the solvation parameter, which is calculated using the absolute value of partial charge for a given atom:

In the above equation (Scheme 1) ASP and QASP are the atomic solvation parameters. The ASP was calibrated using six atom types; while a single QASP is calibrated over the set of charges on all atom types. Since the partial charges calculated with Gasteiger method are 0.619 times lower than the ones calculated with PM6, the QASP parameter in AutoDock 4 and Autogrid source code was reduced by 0.619. Thus, the final QASP parameter used in our AutoDock 4 calculation was 0.00679 (instead of 0.01097) when the PM6 method was used for partial charge calculation.

In Table 3 the results of docking calculations with the new QASP parameter and using PM6 charges on both the ligand and the protein can be seen. 38 out of 53 docking calculations resulted in best energy-lowest RMSD as compared to X-ray. Considering the most populated clusters (and not the lowest energy), 42 first rank and 9 second rank results were observed. In 36 of the 42 successful dockings, the first rank results were "dominant" (at least 60% of the runs resulted in the same cluster) in the docking calculations. In all cases, where the most populated cluster's frequency was above 50 out of 100 runs, the result was accurate (with an RMSD below 2.0 Å as compared to the X-ray structure). It should be emphasized that in all cases where the PM6 charge calculation was used, an accurate docking could be achieved (in one of the clusters there was a result with an RMSD below 2 Å as compared to the X-ray) in contrast to the Gasteiger partial charge calculation dockings, where in eight cases no successful dockings were found (Table 2). The average RMSD of the first rank results is 1.71 Å (1.61 Å without outliers).

**Table 3:** Results of docking calculations using semi-empirical charges on both the ligand and the protein.

PDB code of the complex	RMSD of the first rank result as compared to the X-ray (Å)	Energy rank of the lowest RMSD hit	Frequency rank/frequency of the best geometry	Lowest RMSD (Å)	Estimated free energy of binding (kcal/mol)	Experimental binding energy (kcal/mol)
1AI5	1.22	**1**	**1/100**	0.65	-5.06	-5.08

1AJP	2.73	2	**1/97**	1.07	-4.82	-3.05

1AMW	2.13	**1**	**1/35**	0.87	-5.67	-6.19

1BGQ	1.03	**1**	**1/100**	0.70	-6.50	-11.69

1CBR	1.01	**1**	**1/93**	0.77	-7.42	-10.58

1D3Q	2.77	2	2/40	0.85	-11.47	-8.88

1D3T	2.09	3	**1/60**	0.99	-10.37	-8.73

1DWB	0.72	**1**	**1/100**	0.54	-4.87	-3.98

1FLR	0.39	**1**	**1/100**	0.39	-8.54	-10.98

1GNI	3.01	2	**1/69**	1.01	-6.80	-11.01

1HVJ	1.61	**1**	**1/5**	1.61	-10.45	-14.27

1HVR	1.21	**1**	**1/96**	1.00	-11.67	-12.97

1K4G	2.83	4	**1/89**	1.42	-7.78	-7.99

1KV1	0.6	**1**	**1/100**	0.58	-9.33	-8.10

1LIF	3.1	4	3/17	0.93	-4.51	-9.65

1M0N	1.18	**1**	**1/86**	1.02	-7.65	-3.03

1M0Q	1.03	**1**	**1/80**	0.95	-7.15	-5.30

1OLU	0.89	**1**	**1/89**	0.61	-9.14	-6.01

1Q8T	1.9	**1**	**1/100**	1.41	-7.58	-6.49

1RBP	0.85	**1**	**1/100**	0.56	-8.46	-9.17

1S39	4.29	2	**1/95**	0.33	-4.65	-10.50

1U33	0.86	**1**	**1/43**	0.80	-9.82	-6.28

1ULB	0.7	**1**	**1/100**	0.64	-5.09	-7.20

1UWT	2.35	**1**	**1/53**	0.71	-6.47	-8.14

1X8R	1.26	**1**	**1/100**	0.96	-3.10	-8.36

1XD1	1.81	**1**	2/10	1.30	-15.97	-10.80

1YDT	0.79	**1**	**1/93**	0.45	-8.05	-9.98

1ZC9	2.7	14	2/26	0.96	-6.76	-4.39

2ACK	3.98	4	2/22	0.94	-5.25	-8.97

2BAJ	0.66	**1**	**1/100**	0.55	-12.50	-11.46

2BAK	1.21	**1**	**1/98**	0.64	-14.20	-10.14

2CEQ	0.85	**1**	**1/44**	0.83	-6.23	-9.92

2CET	2.61	**1**	2/15	0.96	-7.76	-10.94

2CGR	0.9	**1**	**1/100**	0.68	-10.05	-9.89

2CPP	1.21	**1**	**1/100**	1.21	-6.26	-8.28

2D3U	0.68	**1**	**1/100**	0.54	-7.11	-9.44

2D3Z	3.86	3	**1/88**	0.63	-7.60	-9.06

2FDP	0.75	**1**	**1/89**	0.68	-14.80	-10.35

2G94	1.75	**1**	3/5	1.45	-8.64	-12.99

2GBP	0.89	**1**	**1/100**	0.87	-5.60	-10.37

2IFB	2.05	2	**1/55**	1.03	-4.99	-7.41

2IWX	0.87	**1**	**1/100**	0.55	-6.76	-9.11

2J77	2.01	**1**	**1/93**	1.60	-7.62	-6.67

2J78	0.83	**1**	2/27	0.73	-6.13	-8.75

2QFU	2.74	4	2/37	0.94	-6.29	-5.70

2QWB	4.35	12	2/15	0.90	-6.64	-3.74

2QWD	2.57	2	**1/67**	0.91	-7.37	-6.62

2R04	1.99	2	2/8	0.92	-8.84	-8.87

2XIS	1.17	**1**	**1/100**	1.14	-6.93	-7.94

2YPI	1.19	**1**	**1/61**	0.78	-2.62	-6.57

3PTB	1.31	**1**	**1/100**	0.74	-5.06	-6.47

4HMG	1.04	**1**	**1/98**	0.85	-6.92	-3.48

7ABP	2.09	**1**	**1/97**	1.16	-5.58	-8.78

	**Average: 1.71 without outliers (rmsd above 4 Å): 1.61**	**38 first hits**	**42 first hits**	**Average: 0.87**		

QASP parameter was modified from 0.01097 to 0.00679 in AutoGrid and AutoDock. The average value of the RMSD between the lowest energy result and experimental structure and the total number of successful first rank predictions (based on lowest energy and highest cluster population, respectively) are indicated at the bottom of the table.

## Discussion

The development of docking software that is able to accurately predict binding geometry and binding energy represents a great challenge in computational chemistry [68,71-73]. In the current study it was explored whether calculation of electrostatic potentials of both the ligands and the proteins using semi-empirical PM6 method increases docking accuracy. With the recent implementation of Mozyme, a PM6 semi-empirical method to the MOPAC2009 software [70], a semi-empirical calculation of partial charges on ligands as well as on larger molecules such as proteins has become possible; therefore, in our study semi-empirical charges were also computed on the protein as well as on the ligand atoms. In the course of the study, 53 experimentally determined protein-ligand complexes were chosen; the ligands were docked back to their host proteins with AutoDock 4 program using 1.) empirical Gasteiger method and 2.) semi-empirical PM6 method for calculating electrostatic potential. The results were then analyzed and compared to the experimentally determined crystal structure in order to evaluate docking accuracy.

**Figure 2:**
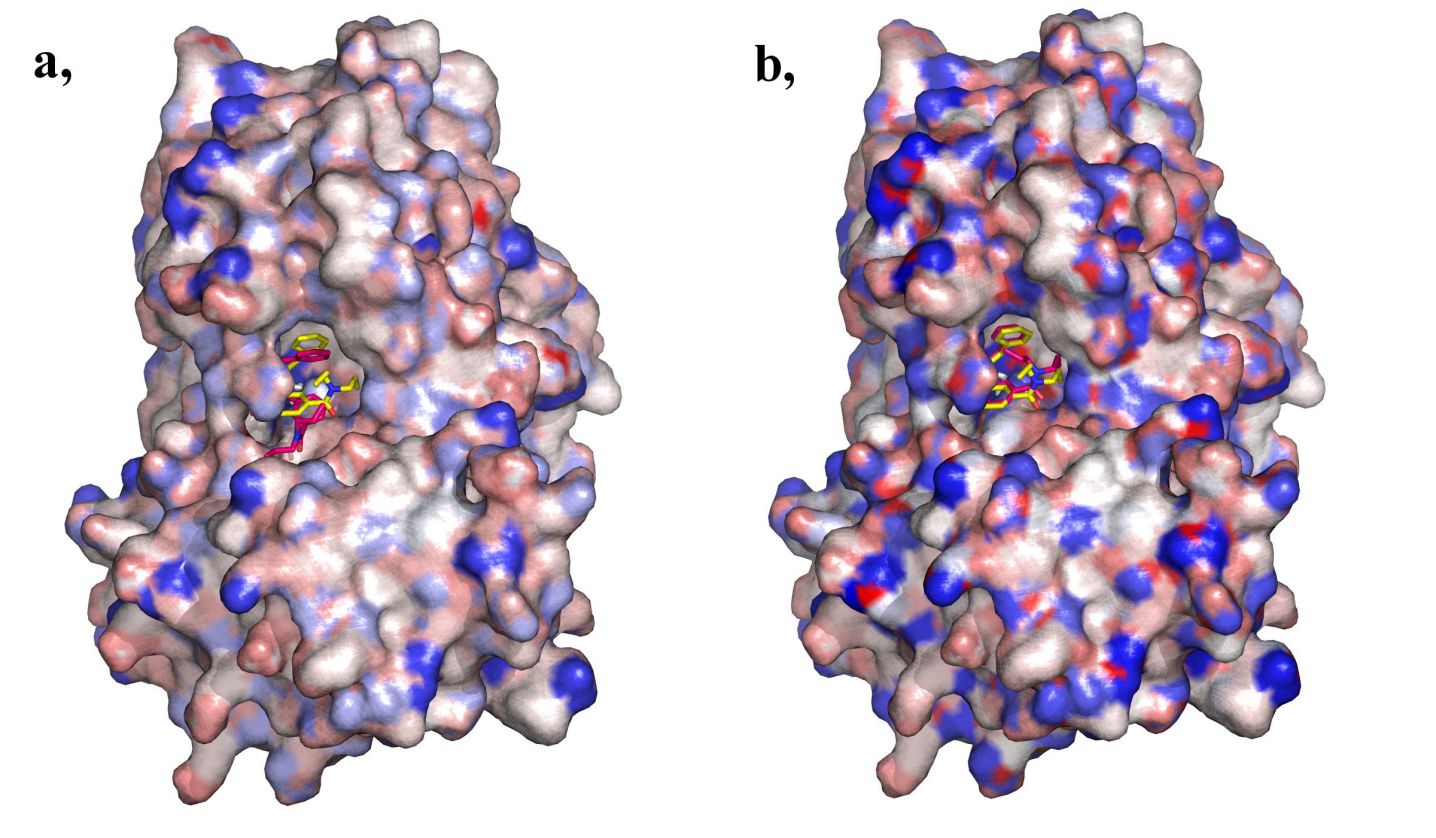
**Best cluster rank docking results of redocking of the PDB entry **2FDP. Protein surfaces are colored by partial charges (**a**, PM6 charges, RMSD from coordinates in PDB: 0.75 **b**, Gasteiger charges, RMSD from coordinates in PDB: 2.35). The darker color of the protein surface colored by PM6 partial charges as compared to the colors of Figure 2b reflects the higher calculated absolute value of semi-empirical partial charges. This "sharper" surface defines the possible binding geometry of the ligand more, than in the case of Gasteiger charges.

It is important to note that AutoDock 4 scoring function - which is used both during and at the end of the dockings thus influencing both the geometry and binding energy estimation - was optimized using the Gasteiger charge calculation method. Since the semi-empirical PM6 calculation method gives higher absolute value for partial charge on a selected atom than the empirical Gasteiger method (Figure 2), each term in the AutoDock equation was carefully considered for expected changes as a result of the semi-empirical method used for partial charge calculation before carrying out docking calculations. Besides the electrostatic term, which is naturally expected to change, absolute values of partial charges are included in the solvation term (**Scheme 1**) [14].

In order to achieve accurate intermolecular energy calculation, the solvation term should not change as a result of the use of different methods for partial charge calculation. Therefore, the extent of change in the absolute value of partial charges using Gasteiger and PM6 methods were analyzed. Our results showed that the partial charges calculated with the Gasteiger method were on average 0.619 times lower than the ones calculated with Mozyme, thus, the QASP parameter was reduced by 0.619. This way the solvation term in the energy calculation of AutoDock 4 has not changed as a result of semi-empirical partial charge calculation.

Our docking results showed that as a consequence of the partial charge calculation with the PM6 method, a dramatic increase was observed in 1.) the number of accurate dockings (i.e. ligand's RMSD is within 2 Å of the actual X-ray structure 2.) population of clusters with the accurate docking result. However, the accuracy of the binding energy prediction has not increased.

The latter finding is not surprising because of several reasons: Although the electrostatic term for a given atom pair is greatly increased when the semi-empirical PM6 method is used for charge calculation compared to Gasteiger charge calculation, this increase is equally present for both negatively and positively charged atoms. Thus, as the positively and negatively charged atoms partly extinguish, and the final electrostatic term summed up for all ligand atoms; the energy related to the electrostatic term will not change to a great extent causing only a minor change in binding energy estimation. Additionally, the weighting constants of these terms in AutoDock 4 were optimized using the Gasteiger charge calculation method; therefore, the final energy calculation is not expected to give a more accurate result using the current weighting constants in the scoring function. Moreover, the method used for partial charge calculation does not influence the torsional entropy term of the equation, which is one of the limiting factors in the accuracy of binding energy estimation in the AutoDock 4 program [74]. In addition, considering the high number of false positives in docking calculations, it is reasonable to assume that in addition to stabilizing contributions, there are destabilizing contributions to ΔG which are in most scoring functions not taken into account [75].

The dramatic change in the number of accurate docking poses is a significant achievement. As discussed above, the electrostatic term for a given atom pair is greatly increased when the semi-empirical PM6 method is used for charge calculation compared to Gasteiger charge calculation and this change is present at different extents in case of each atom type. Thus, a "sharper" electrostatic potential is present using the PM6 method for calculating partial charges (Figure 2) as compared to the Gasteiger charge calculation method. This results in a more pronounced significance of the electrostatic interactions between each atomic pair leading to a more defined protein-ligand complex geometry as compared to the original method where Gasteiger calculation is used.

**Figure 3:**
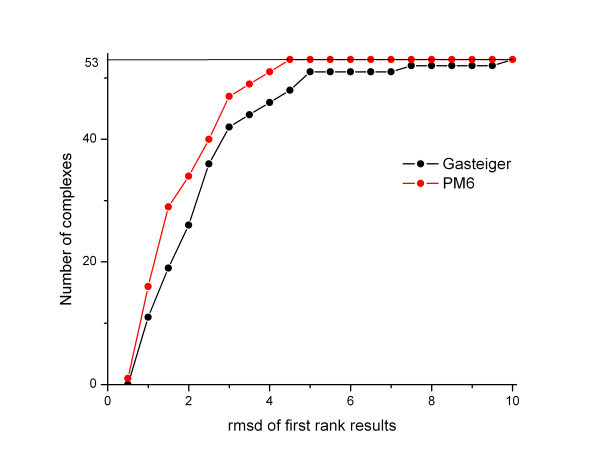
**Performance of the PM6 charge calculation in docking experiments, compared to Gasteiger method**. The graph shows the number of complexes within a given RMSD of the crystallographic structure. In each case, the conformation of the most favorable estimated energy is used as the predicted conformation.

The high population of clusters with the correct geometry with the PM6 charge calculation method is also of great significance (Figure 3). Namely, high cluster population hints at the density of states for a given complex conformation. If the energy of that state does not substantially differ from the lowest binding energy (2.5 kcal/mol is within the standard deviation of the AutoDock 4 force field) then the higher cluster population is indicative of a more probable conformation. This is important information when no experimental data exist as to where the ligand is bound. In conclusion, the use of the PM6 method for calculating partial charges has resulted in a significantly better prediction of docking geometry and in the cluster population of the right docking pose.

Besides accurate binding energy estimation, a measure of docking accuracy is a good geometry, i.e. low RMSD value of the docked ligand as compared to the crystal structure. A recent study [13] reports that developing a scoring function that predicts binding energy with good accuracy is not necessarily achieved by optimizing binding geometry. However, in our study, the correlation coefficient between the experimentally determined and calculated binding energies increased by considering only the hits where the first rank result yielded an RMSD within 2 Å of the actual X-ray structure in docking calculation. Thus, our results indicated that good geometry prediction is indeed prerequisite for accurate binding energy estimation. Although the optimization of AutoDock 4 scoring function was outside the scope of the current study, the fact that significantly more accurate protein-ligand complex geometry prediction is achieved using the PM6 method hints at the possibility of more accurate binding energy estimation using PM6 charge calculation as well.

AutoDockTools, a software used for setting up ligands and proteins for use in AutoDock 4, one of the most popular docking softwares on market [11], is using the empirical Gasteiger method for calculating partial charges for protein and ligand setup. The Gasteiger charge calculation method is based on the partial equalization of orbital electronegativity [17,76]. In the calculation only the topology of the molecule is considered, as only the connectivity of the atoms are included in the calculation. The calculation of electrostatic potential with empirical methods have the advantage of being fast, however, they possess some drawbacks as well: the Gasteiger charge calculation method as opposed to the semi-empirical PM6 method, does not handle inorganic compounds such as metal ions, frequently present in functional proteins. In a study using semi-empirical electronic wave functions like AM1 and PM3 to map experimental dipole moments for a large number of small molecules, the dipole moments were reproduced with a root mean square deviation of 0.3 D [77]. It has been shown based on a large validation set that semi-empirical methods are highly accurate in partial charge calculations and are able to reproduce experimental homo- and hetero-dimer hydrogen-bond energies [16]. Moreover, a number of papers have been published that report increased docking accuracy using the semi-empirical method for partial charge calculation of the ligand atoms [15,22,76]. Indeed, in a recent study comparing several partial charge calculation methods, semi-empirical charge calculation has been shown to increase docking accuracy compared to the use of empirical methods [15]. In that study, partial charge calculation of the ligand alone with the semi-empirical method was sufficient to slightly increase docking accuracy (the protein partial charges were still computed with the Gasteiger method). Quantum mechanical charge calculations on proteins are not very common because of the highly time consuming calculation. One possibility is to consider the effect of the protein binding site on the ligand polarization using quantum chemical methods [19,20]. However, this method still limits the quantum mechanical charge calculations on the ligand and it is difficult to apply when the complex structure is not known. Calculation of quantum mechanical charges using the recent linear scaling Mozyme functionality of MOPAC2009 [23] allows us to calculate quantum chemical charges on protein atoms, as well. Our results suggest that calculation of PM6 charges on protein atoms has an even more profound effect on the docking accuracy.

## Conclusion

In summary, our study explored the effect of the partial charge calculation method on docking accuracy calculated using AutoDock 4 software. To the author's knowledge this is the first systematic docking study where the semi-empirical quantum chemical PM6 method is used for partial charge calculation on the protein as well as on the ligand. Partial charge calculation with the PM6 method has been shown to greatly increase docking accuracy and cluster population of the most accurate docking; however, no increase in the accuracy of binding energy estimation was observed. As a good pose of the ligand seems to be prerequisite for accurate intermolecular energy prediction, the use of the PM6 method presents a great improvement in the accuracy of docking calculations carried out using AutoDock 4. If the PM6 semi-empirical method is used for partial charge calculation, reoptimization of the weighting constants of AutoDock 4 scoring function is needed in order to increase the accuracy of binding energy estimation as well.

## Methods

Crystal structures of the protein-ligand complexes used in this study were obtained from the Brookhaven Protein DataBank http://www.rcsb.org/pdb. When the asymmetric unit was found to differ from the biological unit, the ligand binding site was carefully checked. When the ligand was found to interact with more than one asymmetric units, the biological unit was used in the study (in cases of proteins with PDB code: 1OLU, 2XIS). Experimental binding affinities for the protein-ligand complexes were taken from the PDBBind Database [69]. The proteins and ligands used in this study were all formerly used as a test set in recently published papers [13,68] or were taken from the PDB core set [69]. The structures were chosen in order to meet the following criteria: structurally diverse ligands in complex with a heterogeneous collection of proteins; non-covalent binding between protein and ligand and crystallographic resolution lower than 3.2 Å.

All docking studies described here involved flexible docking of the ligand to the rigid receptor, both of which were derived from the complex crystal structure. Input structures were prepared by using two different methods: (i), using Gasteiger charges for both the ligands and the proteins [17]; (ii), using PM6 charges calculated by MOPAC2009 [70] for both the ligands and the proteins. Briefly, the input structures with Gasteiger charges were prepared as follows: The ligand atom types and bond types were assigned and hydrogens were added using AutoDockTools. Empirical charges were calculated with the method of Gasteiger [17]. For proteins, co-factors, such as HEME and metal ions were kept, and their atom types and bond types were assigned manually. Sulfate, halogens and water molecules were removed. Hydrogens were added in protein residues as well as Gasteiger partial charges using AutoDockTools. Non-polar hydrogens were merged and their charges were added to the heavy atoms. No additional optimization of the protein structures was carried out.

Semi-empirical assignments were performed using the PM6 method by the Mozyme function of MOPAC2009 program [70] integrated in Docking Server http://www.dockingserver.com. Ligand structures with semi-empirical charges were setup similarly as described above, except that in the last step PM6 charges were calculated using MOPAC2009 software. Protein structures were setup as follows: First, water molecules, sulfate, and halogens were removed. Hydrogen atoms were added to the pdb structures using AutoDockTools. The total charge of the protein and partial charges of the atoms were calculated by the Mozyme function of MOPAC2009 software. The calculated partial charges were applied for further calculations.

Docking studies were subsequently performed using Docking Server http://www.dockingserver.com. Docking Server integrates Marvin http://www.chemaxon.com and MOPAC2009 during ligand set up in order to calculate partial charges at a given protonation state and for semi-empirical geometry optimization; and AutoDock 4 is integrated [14] for docking calculation. In cases where protein and ligand partial charges were calculated with the PM6 method, the QASP parameter was modified (QASP = 0.00679) and used in Autogrid 4 and AutoDock 4 during docking calculations (see Results section for detailed explanation).

Briefly, the following parameters were set in Docking Server: Grid parameter files were built and atom-specific affinity maps were constructed using Autogrid 4 [14]. These map files were generated using 60 × 60 × 60 grid points and 0.375 Å spacing, with the maps centered on the experimentally determined center of the bound ligand. Docking simulations for the study were carried out using the Lamarckian Genetic Algorithm. The initial position, orientation, and torsions of the ligand molecules were set randomly, and all rotatable torsions were released during docking. Each docking experiment was derived from 100 different runs that were set to terminate after a maximum of 2,500,000 energy evaluations and had a population size of 250. After each docking calculation, the RMSD between the lowest energy docked ligand pose and the complex crystal structure ligand pose was evaluated.

## Competing interests

The authors declare that they have no competing interests.

## Authors' contributions

Both ZB and EH participated in the design of this study, as well as performing molecular docking and quantum chemical calculations. Both authors participated in manuscript preparation and read and approved the final manuscript.
